# Effect of Isolation Ruminal Yeast from Ruminants on In Vitro Ruminal Fermentation

**DOI:** 10.3390/vetsci12020155

**Published:** 2025-02-11

**Authors:** Krung Wilachai, Pramote Paengkoum, Nittaya Taethaisong, Pirat Thitisak, Kriengsak Poonsuk, Juan J. Loor, Siwaporn Paengkoum

**Affiliations:** 1 School of Animal Technology and Innovation, Institute of Agricultural Technology, Suranaree University of Technology, Nakhon Ratchasima 30000, Thailand; imagine.wilachai@gmail.com (K.W.); iszy.nittaya@gmail.com (N.T.); 2 K.M.P.BIOTECH Co., Ltd. 188/9 Moo 2, Klongtumru, Muangchonburi, Chonburi 20000, Thailand; in-fo@kmpbiotech.com (P.T.); kriengsakpoonsuk@yahoo.com (K.P.); 3 262 Animal Sciences Laboratory, Department of Animal Sciences, College of Agricultural, Consumer and Environmental Sciences, University of Illinois Urbana-Champaign, 1207 W Gregory Dr|M/C 630, Urbana, IL 61801, USA; jloor@illinois.eduansc.illinois.edu; 4 Program in Agriculture, Faculty of Science and Technology, Nakhon Ratchasima Rajabhat University, Nakhon Ratchasim 30000, Thailand

**Keywords:** yeast selection, rumen fermentation, probiotic yeast, ruminants, plate method, yeast strains

## Abstract

In order to obtain high-performing yeast strains from ruminants, it is necessary to select them from species such as beef cattle, dairy cows, goats, and buffalo. A total of 91 isolated yeasts were collected using the standard methods of microbial culture on agar medium followed by streaking on a plate at least three times until pure yeast colonies were formed. The API 20C AUX Kit and sequencing of the D1/D2 domain of the 26S rRNA gene were used to identify the genera *Candida* spp., namely, *C. glabrata* (99% identification), *C. tropicallis* (99%), *C. rugosa* (98%), and *Issatchenkia orientalis* (99%). A total of 12 yeast strains (Dc4, 14, 18; Be1, 2, 7; Bu3, 4, 7; and Go10, 16, 19) were chosen for further analyses. The performance criteria included the ability to tolerate pH values between 3.5 and 7.5, total volatile fatty acids (TVFAs, 0, 0.25, 0.5, 1, 2, and 4% of broth medium), anaerobic growth rate, and in vitro gas production efficiency. First, when all strains were grown at pH values between 3.5 and 7.5, Bu3 and Dc18 performed better than the other strains. Second, at a ruminal pH of 6.5 and a TVFA concentration of between 2 and 4% of the broth medium, strain Bu3 was more resistant than the other strains. Under anaerobic conditions, all strains experienced a decline in viable cell counts when compared with those under aerobic conditions. However, compared to strains Dc14, Be1, Be2, Be7, and Bu3, strain Dc18 exhibited more viable cells under anaerobic conditions in broth medium. The response of strain Dc18 did not differ from those of strains Dc4, Bu4, Bu7, or G16. Strains Be7, Bu3, and Dc18 were used for an in vitro fermentation experiment involving incubation for 2, 4, 6, 8, 10, 12, 24, 36, 48, and 72 h. Three ruminal cannulated dairy cows were used as donors of ruminal fluid. The treatments were run in triplicate. The addition of yeast culture had no effect on gas kinetics, gas accumulation, or the ratio of acetic acid and propionic acid, but led to significantly greater butyric acid concentrations at 24 h of incubation. In conclusion, strain Dc18 isolated from dairy cows is suitable for future studies of probiotic yeast development.

## Simple Summary

Yeast strains are important feed additives that can affect the performance of ruminants. Yeast can be used as a replacement for antibiotics to improve ruminal fermentation and enhance the efficiency of ruminant production systems. Furthermore, the addition of yeast provides nutrients (vitamins, peptides, and growth factors) to the host, which promotes the growth of ruminal microbes. Yeast’s regulation of ruminal microorganisms leads to increased levels of fiber-degrading and lactate-utilizing bacteria, which improves rumen fermentation efficiency and subsequent production capacity. The addition of the yeast culture strain Dc18, isolated from dairy cows, leads to significant differences in butyric acid concentration and is suitable for potential use as a probiotic. This approach not only improves animal performance but also ensures the safety of certain animal products intended for human consumption.

## 1. Introduction

Yeast feed additives have been used as a replacement for antibiotics to improve ruminal fermentation and enhance the efficiency of ruminant production systems. The researchers in [1] reported that the addition of live yeast increased ruminal pH in loose-housed lactating cows. In a review [2], it was noted that the yeast strain *Saccharomyces boulardii* is clinically active, particularly against antibiotic-associated diarrhea pathogens and recurrent Clostridium difficile intestinal infections. However, ruminant nutritionists have shown increasing interest in the use of yeast in animals, although different strains or genera of yeast have various influences on ruminant production. In [3], a supplementary yeast (*Saccharomyces cerevisiae*) culture was studied in fistulated Holstein cows, where higher levels of ruminal anaerobic bacteria—approximately 114 g/cow/d—were found than in the control diet. In another study [4], the yeast strain Saccharomyces cerevisiae NCDC 49 was found to enhance the IVDMD, but *S. cerevisiae* 42 had no effect. Moreover, in [5], different strains of *Saccharomyces cerevisiae* were found to change the percentage of acetate and propionate in the ruminal fluid of cows to different degrees when compared with supplemented cows.

The isolation, identification, and selection of yeast from ruminants, including beef cattle (Be), dairy cow (Dc), goat (G), and buffalo (Bu), were conducted, and the performance of the yeasts was examined. The isolated yeasts were collected using the standard methods of microbial culture on agar medium followed by streaking on a plate at least three times until pure yeast colonies were formed. The API 20C AUX Kit and sequencing of the D1/D2 domain of the 26S rRNA gene were used to identify the genera *Candida* spp., namely, *C. glabrata* (99% identification), *C. tropicallis* (99%), *C. rugosa* (98%), and *Issatchenkia orientalis* (99%). The three strains that exhibited the best performance, Be7, Bu3, and Dc18, were identified using the in vitro gas production technique. In Ref. [6], yeast from the rumen of cows was studied. Yeast belonging to the genera *Candida* and *Trichosporon* were isolated in small numbers (80–1.3 × 10^4^ CFU per gram of fresh rumen content) from the rumen fluid of cattle, being up to 3.5 × 10^3^ CFU/mL when cultured on agar plates at 39 °C. Likewise, in East Greenland, 16 samples of yeast flora obtained from rumen content isolates from musk oxen were examined [7]. The findings showed that various numbers of yeast colonies developed on agar plates incubated at 25 °C, with the counts being up to 1.36 × 10^5^ CFU per gram [8], with the majority of the yeasts belonging to the species *Candida* and *Cryptococcus*.

Recent research carried out in Thailand, a country in Asia considered to be rich in microbial diversity, identified many strains of yeast [9]. In terms of ruminant animals, the researchers in [10] demonstrated that yeasts isolated from the ruminal fluid of dairy cattle can utilize lactic acid when the animals are fed either casava pulp or a highly concentrated strain. The next stage will be to determine whether other strains or species of yeasts can be effective in ruminants, but no information is available regarding either different yeast genera or whether feed additives for ruminants can stimulate fiber degradation. *Candida* spp. have been widely used in the production of biosurfactants from soluble and insoluble carbon sources. For example, the yeast *Candida glabrata* is a biosurfactant that can be produced [11]. However, the authors in [12] suggested that the yeasts *C. tropicallis*, *C. glabrata*, *C. parapsilosis*, and *T. asahii* are able to grow in temperatures above 40 °C and can be considered potentially pathogenic, causing mastitis in dairy cows. The process of selecting yeast strains involves rigorous screening and evaluation based on specific functional traits, such as their ability to improve rumen function and microbiota balance [13]. For instance, Saccharomyces cerevisiae CNCM I-1077 was chosen for its capacity to enhance rumen function through a stepwise screening process involving genetic and functional analyses. The mode of action of yeasts such as *S. cerevisiae* in stimulating rumen fermentation is partly attributed to its respiratory activity, which protects anaerobic rumen bacteria from oxygen damage, thereby promoting a favorable microbial environment [14]. In vitro fermentation is a popular method for evaluating the nutritional value of feed ad-ditives for ruminal fermentation before in vivo experiments [15].

In addition to affecting the microbiota, yeast are able to supply growth factors, organic acids, B-vitamins, and amino acids to ruminal bacteria [16]. In recent years, numerous commercial yeast products have been developed and vary widely in the type of yeast strain. For example, in [17], it was found that the supplementation of 8 × 10^9^ cfu/h/d of *S. cerevisiae* through a ruminal cannula had positive effects on the DM and NDF degradation rate and increased the numbers of ruminal bacteria, fungi, protozoa, and lactate-utilizing bacteria.

A number of studies have isolated yeast from ruminal fluid. For instance, the re-searchers in [10] reported that high concentrations of *Candida* spp., *Trichosporon* spp., and *Picia* spp. isolated from dairy cattle can be used as a probiotic supplement to decrease ruminal lactic production for dairy cattle. In [18], the yeast *Trichosporon asahii* GSY10 was used as a feed supplement in dairy cattle for microbial lipid production. In [19], it was reported that *Candida* spp. improved in vitro ruminal fermentation. The researchers in [20] conducted a study involving crossbred Holstein Friesian steers and found that, compared with *S. cerevisiae* (control), many isolated yeasts obtained from ruminal fluid exhibited high biomass production, cellulase enzyme activity, and cell numbers. However, the authors in [21] reported that the addition of *Canida norvegensis* improved the IVDMD of oat straw, and in [22], it was also observed that the effect of yeast depended on biotic factors such as the strain of the yeast and its viability. Similarly, the experiment described in [23] revealed that *S. cerevisiae* strains differed in their ability to increase the number of viable ruminal bacteria populations in vitro and in vivo. The aim of the present study was to investigate the effect of isolated ruminal yeast and examine the best strains for potential use as a probiotic.

## 2. Materials and Methods

### 2.1. Animal Ethics Statement

The use of animals in this experiment was approved by the National Research Council of Thailand (U1-02632-2559). The Animal Ethics Committee of Suranaree University of Technology approved the experimental protocol (SUT 4/2558), and the animal experiments complied with the ARRIVE guidelines.

### 2.2. Procedures for Isolation and Identification of Yeast Strains

#### 2.2.1. Isolation Step

Yeast was isolated from (1) beef cattle from a slaughterhouse in Ayutthaya province (14°16′52.3′′ N 100°42′29.5′′ E at an elevation of 3.5–5 m above sea level), Thailand; (2) dairy cattle and goats from the Suranaree University of Technology farm; and (3) buffalo at a smallholder farm in Nongboonmark district, Nakhon Ratchasima (14°44′06.6′′ N 102°23′45.6′′ E at an elevation of 187 m above sea level). For the basal feed management, all animals depended on the owners’ operations at the specific farms but relied on commercial concentrate with fresh grass and rice straw. Thus, the basal feed of the beef cattle 2 days before slaughter was rice straw and commercial concentrate; for the dairy cattle, it was rice straw and commercial feed; for the buffalo, it was chopped corn stubble and rice straw; and for the goats, it was a commercial blend.

In the morning before feeding, ruminal fluid was harvested via a stomach tube using the suction technique described by Cuppuccino et al. [24], with the rubber tube and flask being autoclaved at 121 °C three hours before use. The ruminal fluid was immediately placed on ice. Then, 1 mL of ruminal fluid was transferred into 50 mL of autoclaved enrichment broth medium, containing commercial-grade (Titan Biotech Ltd., Delhi, India) glucose, yeast extract, and peptone at 70, 5, and 5 g/L, respectively. The pH was adjusted to 4.5 (Mettler-Toledo AG 8603 Schwerzebach, Switzerland), and the samples were incubated in an incubator shaker at 30 °C with shaking at 150 rpm (New BrunswickTM Innova^®^), (Eppendorf SE · Barkhausenweg 1 · 22339 Hamburg · Germany) for 24 h according to a previously described procedure [9]. Subsequently, the enriched samples were diluted by 5 and spread onto yeast malt agar (YMA) according to the protocol of Boekhout et al. [25]. Briefly, the serial dilution used normal saline to achieve 5 dilutions: 10, 102, 103, 104, and 105 onto the agar plate to count the yeast following the dilutions and after incubation. The culture plates were incubated at 30 °C for 48 h. The yeasts were separated according to colony appearance; i.e., yeast colonies that had formed white-colored circles could be detected visually according to Hesham et al. [26] and were purified using the conventional streaking technique on YMA plates (yeast malt agar, M424-500 g, Himedia laboratory, Dindhori, Nashik, India). The purified yeast was transferred to YMA slant tubes and incubated at 30 °C for 48 h. The samples were then maintained with cryoprotectant medium before storage at −80 °C until the selection step, according to the manufacturer’s protocol.

#### 2.2.2. Identification Step

To characterize the 19 sugars fermented by the yeasts, we used the API^®^ 20C AUX Kit, bioMerieux, Inc., (Durham, NC, USA), which consists of 20 capsules containing dehydrated substrates. In addition, identification was confirmed via the sequencing of domains D1/D2 of the 26S rDNA gene, as described by Lund et al. [6]. Briefly, the domain region was amplified using the primers NL1 (5′-GCATATCAATAAGCGGAGGAAAAG-3′) and NL4 (5′-GGTCCGTGTTTCAAGACGG-3′). The PCR products were sequenced by Humarizing Genomics Macrogen, Geumcheon-gu, Seoul, Republic of Korea.

The isolated yeasts were identified using biochemical methods and molecular sequencing. First, 38 isolates were used for biochemical tests to screen the yeast species. Of those, the following were detected: *Candida glabrata*, *Candida rugosa* (6 isolates), *Candida krusei* (5 isolates), and *Candida tropicalis* (10 isolates). Three isolates could not be identified, and another was identified as *Candida albicans*. Second, molecular sequencing identified eight yeast isolates (Table 1). Four species of *Candida genera* were identified using molecular sequencing with the universal gene on the region D1/D2 domain of the 26S rDNA (98–100% identity).

### 2.3. Selection Procedure

The performance of each of the ruminant yeast sources obtained from stock during the isolation stage was considered. The pure yeast stocks were re-streaked on a YMA medium plate and incubated in an incubator at 30 °C for 48 h for experimental work. A single colony was enough for yeast propagation on the medium when incubated, and this was collected from the agar plate using an aseptic inoculating loop, which was inoculated in the broth medium containing yeast extract, 3.5 g; peptone, 5 g; glucose, 10 g; and deionized water, 1000 mL, according to [4], and incubated at 30 °C for 24 h. A total of 20 yeasts were isolated, 5 each from beef cattle, dairy cattle, goats, and buffalo, and were first tested under a temperature of 39 °C. Following this, three of the best isolates from each animal were chosen for testing of their pH tolerance (3.5–7.5), in an approach adopted from Agarwal et al. [4]; in vitro tolerance to VFA in medium; and yeast propagation on medium under anaerobic conditions.

### 2.4. Tolerance to Organic Acids

Twelve yeast isolates were assessed for their tolerance to organic acids. The main volatile fatty acids (VFAs) in the rumen are acetic, propionic, and butyric acids and were mixed at a ratio of 70:20:10, respectively. They were then added into the broth medium at concentrations of 0, 0.25, 0.5, 1.0, 2.0, and 4.0% (*v*/*v*) and pH-adjusted with 0.1 N of HCL or NaOH to reach the following pH levels: 3.5, 4.5, 5.5, 6.5, 7.0, and 7.5. The tubes contained 7 mL broth. Triplicates from each yeast strain were used in each of the experiments. Live yeast culture (1 mL) was mixed with 50 mL sterile normal saline, and 0.5 mL diluted culture was used for inoculation. The tubes were inoculated at 39 °C for 24 h, and after mixing, the absorbance at 540 nm was recorded to measure the growth of the yeast, as per Agarwal et al. [4]. Each yeast treatment included three replications.

Three replicates from each yeast isolate were used for assessing growth under anaerobic conditions (Menke et al. [27]). A medium consisting of glucose, 20 g; peptone, 20 g; yeast extract, 5 g; and deionized water, 1 mL was placed into tubes (10 mL/tube). Immediately before inoculation, 0.5 mL of a solution containing NaHCO_3_ (10% *w*/*v*), cysteine HCL (0.5%, *w*/*v*), and Na_2_S·9H_2_O (0.5%, *w*/*v*) was added to each tube. The tubes were inoculated with 0.1 mL yeast suspension (on a loopful of a young agar culture suspended in 10 mL deionized water) and incubated in an anaerobic incubator at 39 °C for 24 h. The total viable counts of the culture were measured via the spread plate technique. Colonies were also counted after incubating at 30 °C for 48 h. Three isolates were selected as candidates for the next step.

### 2.5. In Vitro Gas Production Capacity

This experiment was conducted to determine the in vitro gas production at various incubation time intervals. The study was a 4 × 2 factorial as a completely randomized design (CRD) with three replications per treatment. The factors were no-yeast strain (control) or three rumen yeast strains, Be7, Bu3, and Dc18, and two levels (10 and 20%) of the artificial fermentation fluid (Purba et al. [28]). Three ruminally fistulated dairy cows were adjusted for 15 days according to Paengkoum et al. [29] and fed at 2.5% of body weight DM/day on a diet containing rice straw and SUT^®^ (Suranaree university feed factory, Nakhon Ratchasima, Thailand), commercial feed (14% crude protein) (60:40) and served as donors of ruminal fluid. The ruminal fluid was sampled before the morning feed and then placed in warm (39 °C) insulated flasks under anaerobic conditions. All samples were pooled in equal proportions, strained through 8 layers of cheesecloth under anaerobic conditions, and then used immediately.

Single colonies of each ruminal yeast candidate were incubated in 100 mL broth medium, which consisted of yeast extract, 3.5 g; peptone, 5 g; glucose, 10 g; and deionized water to 1000 mL, as per Agarwal et al. [4]. The samples were incubated at 30 °C for 24 h. Afterwards, live yeast culture was inoculated into the strained ruminal fluid in accordance with Newbold et al. [22]. Briefly, artificial saliva was prepared under anaerobic conditions in a water bath at 39 °C with continuous stirring and was mixed with the strained ruminal fluid in a 2:1 ratio (artificial saliva/rumen fluid) to prepare the fermentation solution. Then, 30 mL of buffered fermentation solution was dispensed into 100 mL calibrated Hohenheim glass syringes (pre-warmed in a water bath for 39 °C for 1 h) containing 200 mg substrate consisting of a total mixed ratio (TMR) made by concentrate (SUT^®^ commercial feed 14% of CP) and roughage (rice straw) of 60:40. The live yeast culture concentration and two concentrate levels as treatments were added to the glass syringes. Then, the syringes were incubated in a water bath at 39 °C in triplicate.

During the incubation, the total gas production was measured at 2, 4, 6, 8, 10, 12, 24, 36, 48, and 72 h. Net gas production values were corrected by subtracting blank values from the samples. The cumulative gas production data were fitted to the model described by Ørskov et al. [30], as follows: p = a + b(1 − e^−ct^), where p represents the cumulative gas production at time t, c is the rate of gas production (per hour), and (a + b) is the potential gas production. At 24 h of in vitro incubation, samples of the ruminal fluid were taken and used for estimation. The ruminal pH was determined using a pH meter (HI 9024C; HANNA Instruments, Woonsocket, RI, USA), and VFAs in the filtrates were measured via gas chromatography (Agilent 6890 GC, Agilent Technologies, Santa Clara, CA, USA) using a silica capillary column (30, 250, 0.25 m) Erwin et al. [31].

### 2.6. Statistical Analysis

The results were analyzed as a 4 × 2 factorial in a completely randomized design using the PROC ANOVA and the General Linear Model (GLM) procedures, following the approach described by Steel et al. [32]. For the yeast selection, along with pH tolerance, organic acid resistance, and ability to grow in anaerobic conditions, the analysis was conducted via PROC UNIVARIATE. For in vitro gas production, the following statistical model was used:

Y_ij_ = μ + α_i_ + β_j_ + αβ_ij_ + ε_ij_

where Y = observations, μ = overall means, α_i_ = rumen yeast strain effect (i = no yeast added, Be7, Bu3, and Dc18), β_j_ = doses of yeast (j = 10 and 20% of artificial fermented fluid) effect, αβ = yeast strain effect x doses effect, and ε_ij_ = error. The statistically significant differences among the treatments were determined using Duncan’s New Multiple Range Test (DMRT) at *p* < 0.05, following the approach described by Steel et al. [32].

## 3. Results

### 3.1. Isolation and Identification

**Figure 1:**
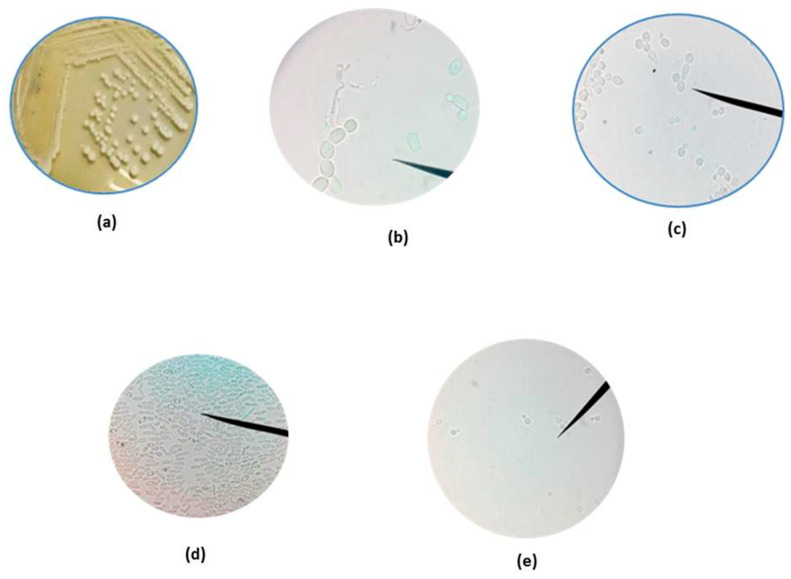
Characteristics of yeast and identified by molecular sequencing that were colonies and budding cell shapes, which are listed as: (**a**) white colonies on agar medium; (**b**–**d**) cell shapes of *Candida tropicalis* as an isolate from buffalo, beef cattle, and goat, respectively; (**e**) *Candida glabrata* isolate from dairy cattle.

Native yeasts were isolated from ruminal fluid obtained from four kinds of ruminants: beef cattle (Bc), dairy cow (Dc), goat (Go), and buffalo (Bu). Although the animals were not treated on each farm, all were robust and had no sign of disease. A total of 91 yeasts were isolated across the ruminant species studied, as follows: 23, 27, 23, and 18 isolates from B, Dc, Go, and Bu, respectively. Although there were differences in cell shape after incubating in broth medium at 39 °C for 24 h, the colonies had features characteristic of yeasts, i.e., the formation of a white circle (Figure 1).

### 3.2. Yeast Selection at Different pH and Organic Acid Concentrations

The pH, short-chain fatty acids, and total volatile fatty acids differed among the yeast strains that were adjusted to the condition of the medium. Consequently, the growth of the yeasts was affected by pH values. The various yeast isolates differed significantly (*p* < 0.01) in terms of their ability to grow under various pH levels (Table 2). All isolates had increased growth when the adjusted pH was 6.5–7.

The yeast grown in the broth medium exhibited different organic acid contents, and the total volatile fatty acids (TVFAs) are shown in Table 3. The isolates Bu3 and Dc18 were more tolerant to TVFAs at all concentrations and had better growth performance than the other isolates (*p* < 0.01) (Table 3).

### 3.3. To Growth in Anaerobic Conditions

**Figure 2:**
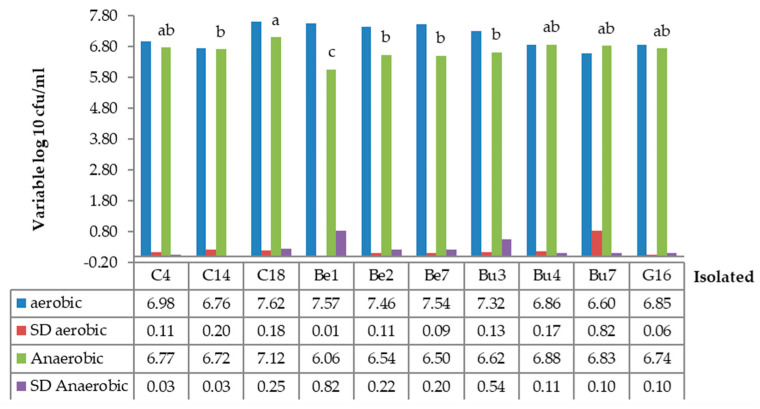
Viable count for different yeast strains incubated under in vitro aerobic (2 replicates) and anaerobic (6 replicates) conditions, in which the incubator was set at 5% O_2_ and 20% CO_2_ for 24 h of incubation. Dc, yeast strain isolated from dairy cow; Be, yeast strain isolated from beef cattle; Bu, yeast strain isolated from buffalo; G, yeast strain isolated from goat. a, ab, b, and c shown significant differences between treatments at (*p* < 0.01).

The growth of the yeast under anaerobic conditions in vitro is shown in Figure 2. For all strains, incubation under anaerobic conditions led to decreased growth when compared with aerobic conditions. It is likely that the propagation of the yeast was limited by the amount of oxygen in the broth and incubator, i.e., 5% oxygen and 20% carbon dioxide. Strain Dc18 exhibited a greater viable cell count than strains Dc14, Be1, Be2, Be7, and Bu3 (*p* < 0.01).

### 3.4. Yeast Supplemented on In Vitro Gas Production Technique

**Figure 3:**
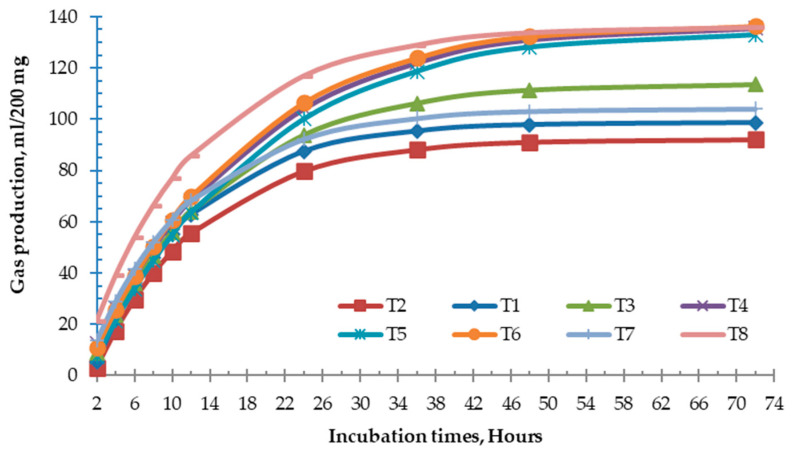
Three replicates of gas cumulative profile of rumen fluid treated for different incubation times: T1 = YC1, T2 = YC2, T3 = YC3, and T7 = YC0 with 10% of fermented fluid; T4 = YC1, T5 = YC2, T6 = YC3, and T8 = YC0 with 20% of fermented fluid.

No interaction (*p* > 0.05) was observed among the main effects, i.e., instantly soluble fraction (a), insoluble fraction (b), and potential extent of gas production (a + b). However, the main effect factor B (dose of live yeast culture) led to a, b, and c values that were significantly different (*p* < 0.01) (Table 4). The volume of gas production at 24 h of incubation was significantly different (*p* < 0.01) among the live yeast strains, and the gas volume increased when added at a high dose (20%) (Table 4, Figure 3).

With regard to the dose of VFAs during incubation are shown in Table 5. the results revealed an interaction between the strain and dose for the acetic acid (C_2_) and acetic acid/propionic acid (C_3_) ratio (*p* < 0.01). In addition, the butyric acid (C_4_) content was significantly different (*p* < 0.01) from the control (YC0, culture alone with no inoculated live yeast).

The effects of live yeast culture on gas accumulation after 2–72 h of incubation are shown in Table 6. Although the gas production at 24 h of incubation was highly significantly different (*p* < 0.01) for the positive control, after 36, 48, and 72 h, inoculation with the yeast strains increased gas production in a dose-dependent manner.

## 4. Discussion

In this study, yeasts from ruminal fluid were able to grow when the pH level was adjusted to 6.5–7. Similar findings were reported in [33], where a rumen pH of 6.8–7 was found to be appropriate for the function of ruminal microbes. Yeast is high in B vitamins, trace elements, unidentified nutrient factors, oligosaccharides, digestive enzymes, amino acids, organic acids, and carbohydrates. In recent years, yeast has been widely used in ruminant production to improve rumen fermentation and animal production performance [34]. In a previous study, it was reported that ruminal microbes differ greatly in their sensitivity to pH, with pH-sensitive species often trying to maintain a close-to-neutral intracellular pH. Ruminal fluid pH is a comprehensive indicator of fermentation and health status. In general, the SARA is characterized by a reduction in ruminal pH below 5.6 [35]. The lower pH in the rumen has a negative influence on the growth and proliferation of microorganisms, causing their death and subsequent inability to release endotoxins, which leads to the dysfunction of the ruminal epithelium [36]. Thus, the strains isolated in this study appear to have adapted to survival in the rumen. Although some yeast strains (e.g., *Saccharomyces cerevisiae*) can tolerate pH levels as low as 2 for 6 h [4], in the presence of autochthonous yeast (*Candida* spp.) isolated from the rumen, they could not propagate at pH 2 and were initially viable at pH 3.5 to 7.5, with pH 6.5 being the optimal condition.

The total volatile fatty acid content of the yeast grown in broth medium is shown in Table 3. Organic acids can pass through the cell wall of the yeast, damaging the cells. The authors in [37] found that yeast growth tended to decrease when TVFAs were added to the medium, likely causing the movement of the organic acids through the cell wall and inducing breakage. These findings agreed with those of previous work demonstrating that a mixture of volatile fatty acids in the broth medium suppressed the growth of yeast [4]. Thus, yeasts such as *Saccharomyces cerevisiae* are unable to grow in the rumen environment because the inherent conditions prevent them from multiplying [38]. It is noteworthy that, in the present study, native yeasts were able to grow in the broth medium even in the presence of VFAs, particularly strains Bu3 and Dc18. Yeast products help regulate ruminal pH by encouraging microorganisms to convert lactate into VFAs, reducing accumulation in the fluid [39]. This result is in contrast to the findings of work carried out in the 1970s [26], demonstrating that yeast growth in the rumen is very limited. The present study underscores the capacity of certain ruminal yeast strains to survive when O_2_ is limited, despite other strains lacking this ability [38].

Gas production is a good predictor of microbial growth and short-chain fatty acid (SCFA) production [40,41]. In vitro fermentation gas production is a key parameter that can be used to reflect the fermentative degree of substrate nutrients by the ruminal microbial community. Generally, more gas production means a higher degree of substrate fermentation [42]. Indeed, some yeast strains (*Candida* spp.) enhance ruminal fermentation and the action of microorganisms on the DM digestibility of fiber. Some studies [18,43] noted that yeast activity may differ depending on the nature and function of the ruminal microbes, and another [10] reported that yeast isolated from the ruminal fluid of cattle exhibited high biomass production, enzyme activity, and cell number in vitro. The same result was found in the current study, although the addition of live yeast strains changed the gas accumulation and VFA content compared to the control. After supplementation with live yeast culture, the activity of the microorganisms in the incubated ruminal fluid was enhanced. It is possible that the live yeast culture was rich in minerals, polysaccharides, small peptides, and digestive enzymes, which promoted the rapid proliferation of ruminal microorganisms and thus increased the degradation rate of the fermentation substrate. The ruminal pH level is related to lactate and VFA concentrations. Additionally, the live yeast culture had a dose-dependent effect on the volatile fatty acid content, although the total VFA content was not influenced by the treatments. These results were similar to those in [44], in which the total VFA and C_3_ increased, while the C_2_ and C_2_/C_3_ ratio decreased with an increasing level of concentration. Ruminal propionate-type fermentation can provide more energy, which has a positive role in maintaining the production performance of animals [45]. As an important energy substrate, glucose plays a vital role in animals’ metabolism. For ruminants, liver gluconeogenesis is the main process of glucose production, and propionate is an important precursor of gluconeogenesis [46]. Therefore, although our experiment was an in vitro study, the results indicate that propionate could provide more energy for the body. A previous study [47] found that using yeast products in dairy calves increased the proportion of *Butyrivibrio* and lactate-utilizing bacteria, leading to higher ruminal propionate and butyrate levels. According to [34], the SARA can negatively impact ruminal epithelial function, leading to inflammation and the decreased absorption of VFA and other nutrients. Moreover, butyrate has the ability to improve the morphology of the ruminal epithelium and increase the surface area of ruminal villi, which are conducive to increasing the ruminal absorption of VFAs [48]. Elevated butyrate concentrations in incubated ruminal fluid may help reduce VFA accumulation. In [49], it was observed that the supplementation of 5 g/d probiotic yeast was adequate to enhance fermentation in the rumen of lactating cattle, as demonstrated by higher VFA concentrations, higher rumen pH, and lower redox potential (Eh) and lactate when compared with non-supplemented control cows. In the same way, diets with more than 50% of concentrate with increasing levels of live yeast improved nutrient digestibility [50]. In contrast, the authors in [51] reported that supplementation with *S. cerevisiae* live cells decreased the acetate level to a greater degree than with *S. cerevisiae* culture.

## 5. Conclusions

The present study revealed that the ruminal environment of dairy cows, beef cattle, buffalo, and goats can sustain the growth of *Candida* spp., including *C. glabrata*, *C. tropicallis*, *C. rugosa*, *C. krusei*, and *Issatchenkia orientalis*. The results obtained via molecular sequencing and in vitro experiments demonstrate that the addition of the live yeast culture Dc18 with 20% of fermented fluid increases gas production and improves both acetic acid (C2) content and the acetic acid/propionic acid (C3) ratio.

## Figures and Tables

**Table 1:** Yeast identified by molecular sequencing of domains D1/D2 of the 26S rDNA gene.

Isolation Sources	Isolated Unknown	GenBank Accession No.	Species	Identify (%)
Beef cattle	Be6	HQ860277.1	*Candida rugosa*	98
	Be7	KU862652.1	*Candida glabrata*	100
Buffalo	Bu3	KY928414.1	*Candida glabrata*	99
	Bu7	EF151501.1	*Candida tropicalis*	99
Dairy cattle	Dc14	EF151501.1	*Candida tropicalis*	99
	Dc18	KM103029.1	*Candida glabrata*	99
Goat	Go19	EU479714.1	*Candida tropicalis*	99
	Go20	EU543672.1	*Issatchenkia orientalis*	99

**Table 2:** Effect of pH (3.5–7.5 n = 216; 12 × 3 × 7-isolate × rep. × pH values) in broth medium on the growth of yeasts.

Isolate	pH Values					
	3.5	4.5	5.5	6.5	7	7.5
Dc4	0.194 ^d^	0.344 ^d^	0.403 ^b^	0.406 ^cd^	0.433 ^c^	0.345 ^c^
Dc14	0.185 ^d^	0.338 ^d^	0.403 ^b^	0.369 ^d^	0.432 ^c^	0.334 ^c^
Dc18	0.357 ^a^	0.449 ^ab^	0.403 ^b^	0.501 ^b^	0.488 ^b^	0.403 ^b^
Be1	0.345 ^ab^	0.406 ^c^	0.373 ^bc^	0.420 ^cd^	0.434 ^c^	0.412 ^b^
Be2	0.348 ^ab^	0.418 ^bc^	0.407 ^b^	0.395 ^cd^	0.417 ^c^	0.320 ^c^
Be7	0.315 ^b^	0.410 ^bc^	0.372 ^bc^	0.428 ^c^	0.436 ^c^	0.401 ^b^
Bu3	0.366 ^a^	0.478 ^a^	0.501 ^a^	0.579 ^a^	0.523 ^a^	0.500 ^a^
Bu4	0.214 ^cd^	0.329 ^de^	0.371 ^bc^	0.423 ^cd^	0.443 ^c^	0.346 ^c^
Bu7	0.238 ^c^	0.357 ^d^	0.395 ^b^	0.396 ^cd^	0.422 ^c^	0.358 ^c^
Go10	0.235 ^c^	0.169 ^f^	0.101 ^d^	0.117 ^e^	0.096 ^e^	0.045 ^d^
Go16	0.203 ^cd^	0.291 ^e^	0.348 ^c^	0.376 ^cd^	0.379 ^d^	0.341 ^c^
Go19	0.238 ^c^	0.189 ^f^	0.115 ^d^	0.113 ^e^	0.103 ^e^	0.064 ^d^
SEM	0.01	0.16	0.19	0.02	0.02	0.02
*p* Value	<0.0001	<0.0001	<0.0001	<0.0001	<0.0001	<0.0001

The letters of superscript in the column mean different highly significant (*p* < 0.01). Dc = yeast strain isolated from dairy cow, Be = yeast strain isolated from beef cattle, Bu = yeast strain isolated from buffalo, and Go = yeast strain isolated from goat. pH (3.5–7.5 n = 216; 12 × 3 × 7-isolate × rep. × pH values), a, b, c, d, e, f in superscripts in a power of a mean value describe significant differences between treatments at (*p* < 0.01).

**Table 3:** Effect of concentration of total volatile fatty acid (TVFAs) in broth medium on yeast growth.

Isolate ^1^	TVFAs (% of Broth Medium)					
	0	0.25	0.5	1	2	4
Dc4	0.357 ^d^	0.359 ^d^	0.378 ^ef^	0.381 ^cd^	0.377 ^d^	0.382 ^cd^
Dc14	0.355 ^d^	0.355 ^d^	0.350 ^f^	0.371 ^de^	0.336 ^e^	0.339 ^d^
Dc18	0.542 ^a^	0.542 ^a^	0.553 ^b^	0.557 ^a^	0.484 ^b^	0.454 ^b^
Be1	0.447 ^bc^	0.447 ^bc^	0.467 ^c^	0.438 ^b^	0.437 ^c^	0.410 ^bc^
Be2	0.419 ^bc^	0.419 ^bc^	0.427 ^d^	0.414 ^bc^	0.379 ^d^	0.355 ^d^
Be7	0.457 ^b^	0.457 ^b^	0.445 ^cd^	0.435 ^b^	0.438 ^c^	0.415 ^bc^
Bu3	0.567 ^a^	0.569 ^a^	0.597 ^a^	0.582 ^a^	0.562 ^a^	0.525 ^a^
Bu4	0.408 ^c^	0.408 ^c^	0.389 ^e^	0.414 ^bc^	0.386 ^d^	0.384 ^cd^
Bu7	0.413 ^bc^	0.413 ^bc^	0.377 ^ef^	0.399 ^bcd^	0.367 ^d^	0.343 ^d^
G16	0.365 ^d^	0.365 ^d^	0.364 ^ef^	0.341 ^d^	0.333 ^e^	0.341 ^d^
SEM	0.07	0.02	0.02	0.01	0.01	0.01
*p* Value	<0.0001	<0.0001	<0.0001	<0.0001	<0.0001	<0.0001

^1^ Dc, yeast strain isolated from dairy cow; Be, yeast strain isolated from beef cattle; Bu, yeast strain isolated from buffalo; and Goat, yeast strain isolated from goat. a, b, c, d, e, and f in superscripts in a power of a mean value describe significant differences between treatments at (*p* < 0.01).

**Table 4:** Effect of live yeast culture and concentrated level supplementation on gas kinetics that was used by an in vitro gas production.

Items	Gas Kinetic ^1^				GP, mL at 24 h
	a, mL	b, mL	c, mL/h	a + b, mL	
^2^ Strain					
YC0	−18.54	124.87	0.14 ^a^	106.33	99.37 ^a^
YC1	−11.58	120.37	0.09 ^b^	108.80	93.93 ^b^
YC2	−13.77	120.69	0.09 ^b^	106.92	90.23 ^c^
YC3	−14.48	121.46	0.09 ^b^	106.98	91.20 ^b^
^3^ Doses, % of fermented fluid					
10	−8.38 ^b^	114.67 ^b^	0.08 ^b^	106.08	87.04 ^b^
20	−20.80 ^a^	129.03 ^a^	0.12 ^a^	108.22	100.33 ^a^
^4^ *p*-Value					
YC vs. Dose	0.150	0.085	0.098	0.120	0.450
YC	0.050	0.061	0.001	0.280	0.001
Dose	0.001	0.001	0.001	0.520	0.001

^1^ a, gas production from dietary soluble fraction; b, gas production from insoluble fraction, but slow release; c, gas production rate constant for insoluble fraction; a + b, potential extent of gas production; GP, gas production at 24 h of incubation (mL/200 mg of DM substrate, 120 g of concentrate, and 80 g rice straw). ^2^ Strain: YC0 = yeast culture, no inoculum; YC1 = yeast culture inoculated with strain Be7 (5.1 × 107 cfu/mL); YC2 = yeast culture inoculated with yeast strain Bu3 (4.3 × 10^7^ cfu/mL); YC3 = yeast culture inoculated with yeast strain Dc18 (4.1 × 10^7^ cfu/mL); 1000 mL yeast culture contained 3.5 g of yeast extract, 5 g of peptone, and 10 g of glucose. ^3^ Doses of supplementation: 10% artificial rumen fluid, 20% artificial rumen fluid. ^4^ YC vs. Dose = interaction of yeast strain and level of supplementation, YC = main effect A, Level = main effect B. a, b, c in superscripts in a power of a mean value describe significant differences between treatments at (*p* < 0.05).

**Table 5:** Effect of live yeast culture (effect factor A) and doses added (effect factor B) on volatile fatty acid (VFA) content.

Items	VFA (%)				Total VFA (mmoL/L)
	C2	C3	C4	C2:C3	
^1^ Strain					
YC0	59.22 ^a^	29.09	11.70 ^b^	2.04	105.65
YC1	57.13 ^b^	28.45	14.42 ^a^	2.01	104.72
YC2	57.56 ^b^	27.83	14.61 ^a^	2.08	110.02
YC3	58.15 ^ab^	27.80	14.05 ^a^	2.10	113.13
Doses, % of fermented liquid					
10	56.61 ^b^	28.64 ^a^	14.74 ^a^	1.98 ^b^	105.60
20	59.20 ^a^	27.79 ^b^	13.01 ^b^	2.14 ^a^	111.66
*p* Value					
YC vs. Dose	0.001	0.05	0.05	0.001	0.251
YC	0.001	0.10	0.001	0.353	0.451
Dose	0.001	0.035	0.001	0.001	0.300

^1^ Strain; YC0 = yeast culture, no inoculum, YC1 = yeast culture inoculated with strain Be7 (5.1 × 10^7^ cfu/mL), YC2 = yeast culture inoculated with yeast strain Bu3 (4.3 × 10^7^ cfu/mL), YC3 = yeast culture inoculated with yeast strain Dc18 (4.1 × 10^7^ cfu/mL); 1000 mL yeast culture contained 3.5 g of yeast extract, 5 g of peptone, and 10 g of glucose. Doses of supplementation: 10% artificial rumen fluid, 20% artificial rumen fluid. YC vs. Dose = interaction of yeast strain and level of supplementation, YC = main effect A, Level = main effect B, a, b in superscripts in a power of a mean value describe significant differences between treatments at (*p* < 0.05).

**Table 6:** Effect of live yeast culture (main effect factor A) and doses added (main effect factor B) on gas cumulative at 2–72 h of incubation.

Items	Gas Accumulates, mL/ 200 mg of Substrate									
	H2	H4	H6	H8	H10	H12	H24	H36	H48	H72
^1^ Strain										
YC0	11.70	34.20	51.00	63.61 ^a^	73.12 ^a^	80.33 ^a^	99.37 ^a^	114.66	118.47	119.96
YC1	8.75	21.87	39.30	50.71 ^b^	60.16 ^b^	68.00 ^b^	93.93 ^b^	91.74	94.46	95.37
YC2	5.40	21.38	34.75	46.00 ^c^	55.31 ^c^	64.39 ^b^	90.23 ^c^	114.06	121.15	124.49
YC3	5.40	25.50	35.56	46.96 ^bc^	60.16 ^bc^	80.33 ^a^	91.20 ^c^	121.24	130.18	134.62
^2^ Dose, % of fermented fluid										
10	8.39	22.53	34.48	44.61 ^b^	53.22 ^b^	60.56 ^b^	87.0 ^b^	105.59	110.77	113.07
20	7.22	28.84	45.82	59.00 ^a^	69.30 ^a^	77.38 ^a^	100.33 ^a^	114.49	120.92	123.91
^3^ *p* Value										
YC vs. Dose	**	**	*	0.07	ns	ns	ns	**	**	**
YC	**	**	**	**	**	**	**	**	**	**
Dose	*	**	**	**	**	**	**	**	**	**

^1^ Strain; YC0 = yeast culture, no inoculum, YC1 = yeast culture inoculated with strain Be7 (5.1 × 10^7^ cfu/mL), YC2 = yeast culture inoculated with yeast strain Bu3 (4.3 × 10^7^ cfu/mL), YC3 = yeast culture inoculated with yeast strain Dc18 (4.1 × 10^7^ cfu/mL); 1000 mL of yeast culture contained 3.5 g of yeast extract, 5 g of peptone, and 10 g of glucose. ^2^ Doses of supplementation: 10% artificial rumen fluid, 20% artificial rumen fluid. ^3^ YC vs. Dose = interaction of yeast strain and dose of supplementation, YC = main effect A, Dose = main effect B, a, b, and c in superscripts in a power of a mean value describe significant differences between treatments at * *p* < 0.05, ** *p* < 0.01, ns = not significant.

## Data Availability

Data are contained within the article.
